# A Pattern-Based Approach for Detecting Pneumatic Failures on Temporary Immersion Bioreactors †

**DOI:** 10.3390/s19020414

**Published:** 2019-01-20

**Authors:** Octavio Loyola-González, Miguel Angel Medina-Pérez, Dayton Hernández-Tamayo, Raúl Monroy, Jesús Ariel Carrasco-Ochoa, Milton García-Borroto

**Affiliations:** 1 Tecnologico de Monterrey, School of Engineering and Science, Vía Atlixcáyotl No. 2301, Reserva Territorial Atlixcáyotl, Puebla 72453, Mexico; 2 Tecnologico de Monterrey, School of Engineering and Science, Carretera al Lago de Guadalupe Km. 3.5, Atizapán, Estado de México 52926, Mexico; migue@tec.mx (M.A.M.-P.); raulm@tec.mx (R.M.); 3 Centro de Bioplantas, Universidad de Ciego de Ávila, Carretera a Morón km 9, Ciego de Ávila 69450, Cuba; daytontamayo@gmail.com; 4 Instituto Nacional de Astrofísica, Óptica y Electrónica, Luis Enrique Erro No. 1, Sta. María Tonanzintla, Puebla 72840, Mexico; ariel@ccc.inaoep.mx; 5 Instituto Superior Politécnico José Antonio Echeverría, Calle 114 No. 11901, Marianao, La Habana 19390, Cuba; mgarciab@ceis.cujae.edu.cu

**Keywords:** temporary immersion bioreactor, failure detection, contrast patterns

## Abstract

Temporary Immersion Bioreactors (TIBs) are used for increasing plant quality and plant multiplication rates. These TIBs are actioned by mean of a pneumatic system. A failure in the pneumatic system could produce severe damages into the TIB. Consequently, the whole biological process would be aborted, increasing the production cost. Therefore, an important task is to detect failures on a temporary immersion bioreactor system. In this paper, we propose to approach this task using a contrast pattern based classifier. We show that our proposal, for detecting pneumatic failures in a TIB, outperforms other approaches reported in the literature. In addition, we introduce a feature representation based on the differences among feature values. Additionally, we collected a new pineapple micropropagation database for detecting four new types of pneumatic failures on TIBs. Finally, we provide an analysis of our experimental results together with experts in both biotechnology and pneumatic devices.

## 1. Introduction

Temporary Immersion Bioreactors (TIBs) are used for increasing plant quality and plant multiplication rates [1,2,3,4,5]. TIBs are quite popular in both industry and academia, because they usually reduce production costs [4]. Roughly, a TIB works by scaling-out automated micropropagation of plants under standardized conditions, excluding microbial contamination. There are different types of TIBs, but the most popular ones [1] use a pneumatic system drive to execute periodic tasks for guaranteeing both efficiency and efficacy. (Experts in biotechnology take efficacy to mean high plant growing rate and high quality plants, while efficiency indicates low production cost.) Any problem in the pneumatic system of a TIB may produce severe damages over the plant micropropagation. Consequently, the whole process of micropropagation would be aborted due to the failures during plant immersion into the nutrient medium [6]. Therefore, failure detection in a TIB is very important to avoid any problem during plant micropropagation.

The failure detection in a pneumatic system of a TIB often is a class imbalance problem, because the number of failures is small compared with the number normal immersion process [6]. Failure detection can be tackled using a one-class classification approach, where the data are labeled as normal for normal immersion and abnormal for failures.

Contrast pattern-based classifiers have been successfully used in practical problems, such as structural alerts for computational toxicology [7], gene transfer and microarray concordance analyses [8], characterization for subtypes of leukemia [9], classification of spatial and image data [10], classification of lymphomas [11] identification of real objects [12], discovering pattern in dynamic databases [13], gene expression profiles [14], characterization of click-stream sequences from e-commerce websites [13], detecting pneumatic failures on TIBs [6], and prediction of heart diseases [15]. Thus, contrast pattern-based classification is suitable for detecting pneumatic failures in a TIB, since:

(i) It has reported good classification results for this kind of problem [6].
(ii) It has reported outstanding classification results in class imbalance problems, overcoming other state-of-the-art classifiers designed for class imbalance problems [16,17].

In addition:

(iii) Contrast patterns are expressed in a language close to that used by human experts in the application domain [14,18].
(iv) They can be introduced in an easy way into a programmable logic controller (PLC), which is used to manage a TIB [6,19].

In this paper, we present a classifier based on contrast patterns for detecting pneumatic failures on TIBs. Preliminary results of this study are reported in a conference paper [6]. We successfully tested our classifier using eight real-world databases of pineapple plants proposed in [6] and a new real-world database of pineapple plants collected in our experiments.

The main differences between this paper and the method proposed in [6] are the following: we include a significantly larger number of classifiers, of different nature; we introduce a new real-world database of pineapple plants for pneumatic failure detection; we extract new features from the original dataset; we detect four new types of failures; we include an analysis together with experts in both biotechnology and pneumatic devices; from this analysis, we have identified the most frequent patterns describing failures in TIBs; and, finally, we include an explanation about the classification results obtained by our proposal in a real-world experiment of micropropagation of pineapple plants by using TIBs.

The main contribution of this paper is a contrast pattern-based classification approach for TIBs pneumatic failure detection. Our classifier allows creating an explanatory and accurate model that forewarns expert practitioners about six types of TIB failures. A further contribution of this paper is to provide an interpretation of the classification results, issued by biologists, regarding the time intervals for immersing periodically the plants into the nutrient medium in a real-world experiment of micropropagation of pineapple plants using TIBs.

The rest of the paper has the following structure: Section 2 explains how TIBs work and the workings of a pneumatic system. Section 3 details the materials and methods used for detecting pneumatic failures on TIBs where the tested databases are explained. Section 4 presents our results as well as a detailed discussion, including interpretations issued by pneumatic and biotechnologist experts. Finally, Section 5 provides conclusions and future work.

## 2. Temporary Immersion Bioreactors

Manual labor is the major cost in plant propagation by tissue culture. This is because a biologist must manipulate each individual shoot, which may yield a high level of contamination. To avoid this problem, Temporary Immersion Bioreactors (TIBs) were introduced [20]. TIBs work by taking advantage of the effects of a liquid medium over the culture. They help to attain high quality plants and high plant growth rates, while reducing production costs. TIBs can be either mechanically agitated (ma-TIBs), or pneumatically agitated (pa-TIBs) [1,21]. In this paper, we focus on pa-TIBs because we could not collected data from ma-TIBs for testing our proposal.

TIBs are commonly used because they yield higher multiplication rates than other techniques. For example, in coffee (*Coffea arabica* or *canephora*), the multiplication coefficient, using a semi-solid medium, ranges from six to seven every three months; however, using a TIB, a similar multiplication coefficient can be obtained in only five weeks [20]. (The number of total outbreaks per explant was quantified. The equation for computing the multiplication coefficient is: MC = Number of total outbreaks/Number of initial outbreaks.) Using TIBs is very attractive, due to the following reasons [22,23]:

(i) They avoid the continuous immersion of the culture into the liquid medium; otherwise, this would adversely affect plant growth and morphogenesis.
(ii) They avoid an adequate oxygen transfer and sufficient mixing; otherwise, this would affect the plant growth.
(iii) They facilitate both automation and changing the liquid medium.
(iv) They minimize human intervention, and so the chances of contamination; otherwise, this would increase production costs.

**Figure 1:**
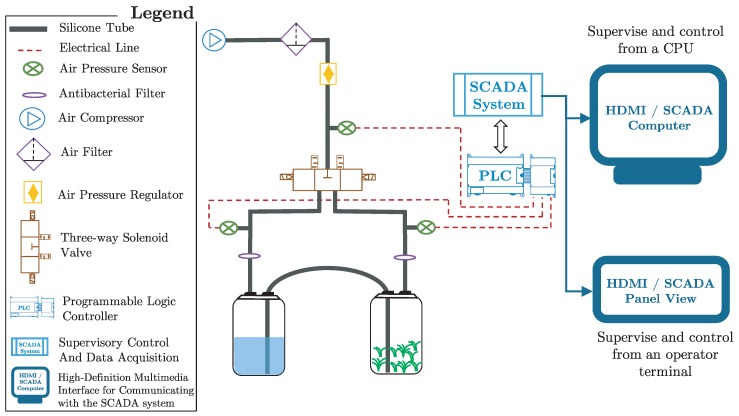
Temporary immersion bioreactor diagram.

At the Centro de Bioplantas (www.bioplantas.cu), there is a pneumatically agitated bioreactor, which is formed by two transparent glass (or plastic) containers, auto-clavable silicone tubes, hydrophobic air filters, electric valves, and an air compressor [1]. This TIB (see Figure 1) has one container for plant growing and another one for liquid medium. Both containers are connected via silicone tubes where air flow is sterilized as it passes through hydrophobic filters.

**Figure 2:**
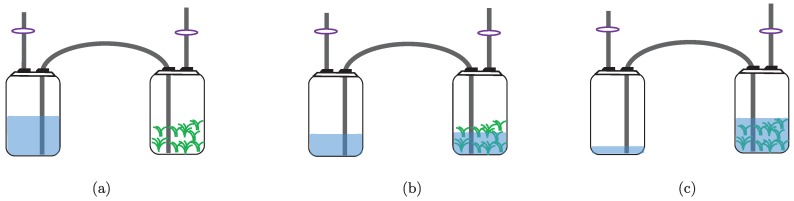
Operating cycle of the TIB: (**a**) non-immersed stage; (**b**) beginning of the immersed stage; and (**c**) immersed stage.

**Figure 3:**
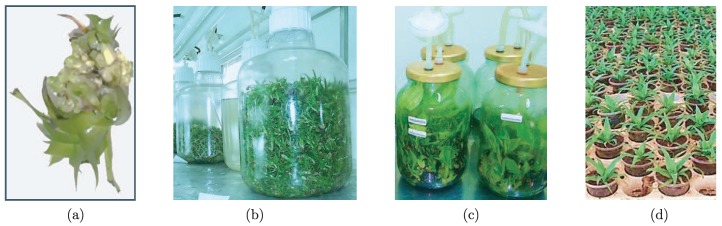
Phases inside and outside TIBs: (**a**) multiplication: typical shoots; (**b**) elongation: commonly produced into containers of high volume; (**c**) rooting: previous phase before carrying a plant to field; and (**d**) acclimatization: final phase where plants adapt to the environment (i.e., outside the TIB).

The immerse stage begins when an air compressor pushes the medium from one container to the other by mean of air pressure. Then, air flow is reversed to withdraw the medium from the culture container (see Figure 2). For doing this, three-way solenoid valves (the valve model used is SY7440 from SMC manufacturer; see the following url for more information: http://www.smcpneumatics.com/SY7440-5DZ.html) provide on/off operation, where the frequency and length of the immersion period is controlled using a programmable logic controller (the PLC model used is MASTER-K120S from LG manufacturer; see the following url for more information: http://foster.pl/en/index.php?id=c05_02) (PLC) [19] connected with a supervisory control and data acquisition (SCADA) system through the modbus protocol [24]. This type of TIB has reported high growth rates in different kinds of plants, e.g., plantain, pineapple, and sugarcane [2,3,4,25,26,27,28,29]. There are three main phases to carry out inside TIBs (in vitro): multiplication, elongation, and rooting. After, the plants are taken to a final phase: acclimation (ex vitro), which is carried out outside TIBs (see Figure 3).

The use of TIBs at the first stages of pineapple propagation enables precise control of plant growth, increases the rate of plant multiplication, and decreases space, energy and labor requirements for pineapple plants in commercial micropropagation [3]. At Centro de Bioplantas, the pineapple culture is one of the most common uses of TIBs [1,2,3,4]. In this research center, the stage reporting higher growth rate in pineapple plants is the multiplication stage.

According to Escalona et al. [1] and Aragón et al. [25], to obtain the best multiplication rate in pineapple plants, using a TIB, shoots should be immersed for two minutes every three hours during 42 days. Depending on the volume (according to Escalona et al. [1], a container of 1000 mL of liquid medium is used for obtaining the time of every operating cycle of immersion) of the container for liquid medium, the full operating cycle of immersion can take, approximately, four minutes: one minute for pushing the medium from one container to the other (see Figure 2), two minutes of immersion, and one minute for withdrawing the medium from the culture container.

During 42 days, pineapple plants are, approximately, 6.4 h inside the liquid medium (immersion time) and the remaining time outside of it. Commonly, at night, in the Centro de Bioplantas, there are no experts around for supervising every experiment using TIBs. Consequently, if during this time interval a failure arises in the TIB (e.g., an obstruction (the most frequent obstruction is produced by waste of plant material) into the silicone tube connecting the containers), then the process can be aborted.

When a failure is detected in the pneumatic system of a TIB, biotechnologist experts inspect all the system and carry out tasks according to solve the failure, for example, replace clogged lines or filters. All tasks executed for solving any problem in a TIB should follow strict aseptic operations. In some cases, obstructions are produced by the waste of plant material; consequently, experts expect that the PLC can detect this obstruction and blow the air with more pressure for withdrawing it and as a result reduce the expert interventions for solving this type of failures.

### The Contrast Pattern-Based Approach for Detecting Pneumatic Failures on TIBs

As a first step for solving this problem, an approach for detecting pneumatic failures on TIBs was proposed in [6]. This approach uses a contrast pattern-based classifier (HeDex proposed by Kang and Ramamohanarao [30]) for extracting patterns describing pneumatic failures on TIBs. HeDex is based on a decision trees ensemble using the Hellinger distance [31] as a decision tree splitting criterion. Hellinger distance is unaffected by the class imbalance problem because it rewards those candidate splits that maximize the true failure rate while minimizing the false failure rate.

A pattern is an expression defined in a certain language that describes a collection of objects [17,32,33]. Usually, it is represented by a conjunction of relational statements, each of the form: $\left[f_{i}\;\#\;v_{j}\right]$, where $v_{j}$ is a value in the domain of feature $f_{i}$ and # is a relational operator from the set $\left\{=,\neq ,\leq ,>\right\}$. For example, [Root_rot>5]∧[Green_leaves≤5]∧[Stem_canker=“Yes”]∧[Leaf_spot=“Several”] is a pattern, written in a logical form, that describes a collection of plants suffering from bacterial canker. Let *p* be a pattern and *D* be a dataset; then, the support of *p* is a fraction resulting from dividing the number of objects in *D* described (support) by *p* by the total number of objects in *D*. In this way, a contrast pattern (CP) is a pattern describing significantly more objects from a class than from the remaining problem’s classes [33,34,35].

The pneumatic failures detected by Loyola-González et al. [6] were regarding bad air pressure, specifically into: the central distribution line, the plant container, and the liquid medium container.

To detect these kinds of failures in [6], we used eight databases of micropropagation of pineapple plants. Each database contains objects with information about a time interval of 70 s where the TIB pneumatic system was powered on. Objects can belong either to the class *failure*, or not. An object of the class *failure* represents the occurrence of a problem during the immersion time. It is worth noticing that these eight databases are all imbalanced. These databases present different class imbalance ratio (IR) [36], ranging from 9.48 to 21.45.

In [6], HeDex [30] yielded the best classification result in the detection of pneumatic failures on TIBs. In addition, the authors analyzed the patterns extracted from HeDex, concluding that it found patterns, with high support, describing the failure class. The patterns are the following:

(i) Air pressure into the central distribution line is lower than or equal to 0.104 bar.
(ii) Air pressure into the plant container is lower than or equal to 0.136 bar and air pressure into the central distribution line is greater than 0.127 bar.

The main drawback of Loyola et al.’s approach [6] is that their patterns describe a failure without identifying where in the associated TIB the failure stems. Through a thorough discussion of this with biologists, the main users of TIBs, we know that they prefer to fully pinpoint a failure occurrence. For example, for an expert, it is mandatory to know whether a failure has arisen in the fitting of the filter or in the fitting of the siphon, or even whether this failure is in the liquid medium container or in the plant container. The main reason for this is that some failures are less affordable than others. For example, a failure in the fitting of the siphon in the liquid medium container only produces air leak but it does not affect the whole process if it is looked after in a short interval of time. However, if a failure is detected in the fitting of the filter in the liquid medium container, then it should be given the highest priority because it could spill all the liquid medium out of the container. In a different vein, the method of Loyola et al. was tested only against four other, contrast pattern-based, rival methods. Thus, further and thorough experimentation, including a greater number of heterogeneous classifiers, is required for corroborating that the contrast pattern-based approach outperforms others for solving this type of problem.

**Figure 4:**
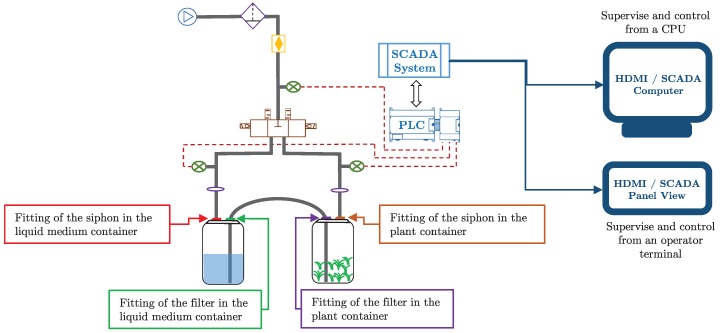
The new failures that we propose to detect in the TIBs. SCADA refers to Supervisory Control And Data Acquisition; PLC refers to Programmable Logic Controller; and HDMI refers to High-Definition Multimedia Interface, which is used for communicating with the SCADA system.

Then, based on these reasons, we propose to create an experimentation that includes classifiers based on different approaches, e.g., one-class, contrast patterns, logistic regression, and decision trees, among others. In addition, we propose to detect the following specific failures (see Figure 4):

(i) Pressure failure on the fitting of the siphon into the liquid medium container.
(ii) Pressure failure on the fitting of the filter into the liquid medium container.
(iii) Pressure failure on the fitting of the siphon into the plant container.
(iv) Pressure failure on the fitting of the filter into the plant container.
(v) Pressure failure on the fitting of the siphon for both liquid medium and plant container.
(vi) Pressure failure on the fitting of the filter for both liquid medium and plant container.

Testing different approaches based on patterns, we can create an explanatory model for detecting the above-described failures on TIBs. By doing so, we can help application domain experts to obtain high plant growing rates, high quality plants, and low production costs.

## 3. Materials and Methods

In this section, we present all materials and methods used in this paper. To show that using contrast pattern-based approaches allows obtaining good classification results for detecting pneumatic failures on TIBs, we contrasted several supervised classifiers of different paradigms.

We executed two types of experiments using all the selected classifiers: (i) using our proposed database where there were six types of failures to be identified; and (ii) using the databases proposed in [6] and our proposed database but using a two-class approach (see Section 3.1).

This section has the following structure: first, in Section 3.1, we show all characteristics of the databases as well as the validation procedure executed in our experiments. After, in Section 3.2, we present a brief explanation of the selected classifiers. Next, in Section 3.3, we show the measures used for evaluating the performance of the classifiers. Finally, we describe the statistical tests used for comparing classification results in Section 3.4.

### 3.1. Datasets and Data Pre-Processing

In this paper, we introduce a new micropropagation pineapple database for detecting pneumatic failures in TIBs. This database contains 951 objects. We extract 70 features for every air sensor of the pneumatic system on a TIB. Every feature represents the air pressure of a single sensor for an instant of time (every sensor measures the air pressure during 70 s with intervals of one second). The pneumatic system contains the following three air sensors: a sensor within the liquid medium container, a sensor within the plant container, and a sensor in the central distribution line (see Figure 1).

For every consecutive (in time) measures of every air sensor, we have also extracted the variation of air pressure. This allows us to have information about the increasing (or decreasing) speed of the air pressure. Our hypothesis here is that, in the case of a failure, the speed of change of air pressure will be different to that computed in normal conditions.

It is important to highlight that the feature *deltaPfv1* is the difference between the last feature *ps* and the first feature *pfv*. It tries to capture the difference between the measure of the sensors *ps* and *pfv*. In the same vein, the feature *deltaPfll1* is the difference between the last feature *pfv* and the first feature *pfll*. It tries to capture the difference between the measure of the sensors *pfv* and *pfll*. These features proved to be useful as well as they appear in several patterns of high support so they have an important role in the performance of classification.

Every object in the dataset is labeled with one of the following classes: normal, fault 1, fault 2, fault 3, fault 4, fault 5, and fault 6. Accordingly, each fault class denotes a different failure occurring during the immersion time (see Table 1).

For each type of failure, initially, we collected 200 objects, but, after a correlation analysis, we removed duplicate and noisy objects for obtaining a representative database. Consequently, we obtained a different number of objects for each type of failure, as shown in Table 1.

**Figure 5:**
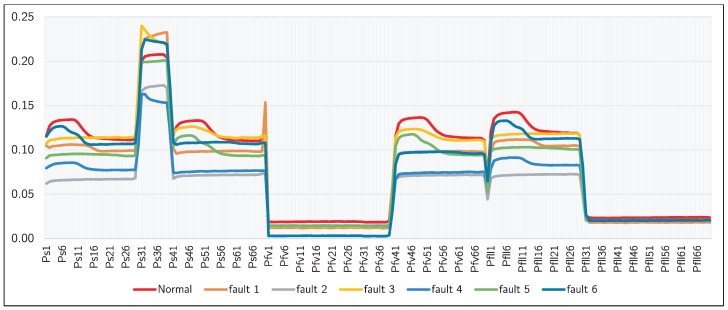
A representation based on time series for the average data of normal class and each of the six faulty classes.

Figure 5 shows a representation based on time series for each class detailed in Table 1. This figures plots a time series taking into account the 70 s with intervals of one second for each of the three sensors used in the TIB. In this figure, we can see that all class of the problem have a similar behavior among them, which makes this one a hard problem for detecting both normal and failure behavior.

In addition, we used in our experiments the databases introduced in [6]. The aim was to corroborate that our proposal can detect other kinds of pneumatic failures.

Table 2 shows, for each database introduced in [6], the database name, the number of objects belonging to the minority (failure) class (#Objects_Min), the number of objects belonging to the majority class (#Objects_Maj), and the class imbalance ratio (IR).

We partitioned all databases using five-fold and distribution optimally balanced stratified cross validation (DOB-SCV) [37] with the goal of avoiding problems about data distribution imbalanced databases [38].

### 3.2. Tested Supervised Classifiers

In this section, we show the different supervised classifiers selected in this paper for testing the performance in the detection of a pneumatic failure in a TIB. The classifiers’ parameters were configured following their authors’ recommendation. Table 3 shows, for each tested classifier, its abbreviation used throughout the paper, its full name, the approach followed for the tested classifier, and its reference.

In our experiments, we used kNN, AB.M1, J48, LogReg, MLP, NBayes, RF, and SVM by using Weka Data-Mining software tool [50]. On the other hand, TreeBagger, B-TPMiner, OCKRA, and PBC4cip were provided by their corresponding authors.

### 3.3. Evaluation Methodology

To assess the performance of the selected classifiers, we used the following measures, because these measures are suitable for class imbalance problems.

**AUC:**  Area Under the receiver operating characteristic Curve [51]: AUC evaluates the true failure detection rate ($TP_{rate}$) versus the false failure detection rate ($FP_{rate}$). This measure is the most used for assessing the classification results in class imbalance problems because, as stated by Japkowicz [52], it measure is not affected by subjective factors, unlike G-mean [53] and F-Measure [54]. In addition, AUC is insensitive to changes in the distribution of the training dataset, which make it suitable for class imbalance problems.
**ZFP:**  Zero-False Positives is another important measure for assessing the classification results. ZFP measures the number of true failures (TP) when no false failure (FP) is detected; i.e., the value of TP when FP = 0. The higher is the ZFP value, the more reliable is the failure detection system. The main reason for using this measure is to evaluate the number of false positive alerts in a short time interval of time [48], which can be disturbing for users receiving continuously false positive alerts.

We computed these performance indicators from Receiver Operating Characteristic (ROC) curves according to Fawcett [55]. In addition, we averaged the results of the five-fold cross validation (5-FCV) performance indicators for each database.

### 3.4. Statistical Tests

It is common in an experiment to check whether the classification results produced by the selected classifiers are statistically different. Consequently, we applied the Friedman’s test (a nonparametric test) and after, we performed the Finner’s procedure (a post-hoc procedure), as suggested in [56,57].

The Friedman’s test is used to know whether there are significant statistical differences among the classification results, but it is not able to determine which results have statistical differences among them. Hence, Shaffer’s static procedure (as a post-hoc procedure), suggested by García and Herrera [57], is used for determining which results have statistical differences among them. Although there exist other important post-hoc procedures, e.g., Nemenyi, Holm, and Bergmann–Hommel procedures, we selected the Shaffer’s static procedure because it is more powerful and less computationally expensive than others well-know post-hoc procedures [57].

It is important to highlight that all statistical tests were executed with a level of significance α=0.05, as proposed in [56,57]. We used the KEEL software suite [58] for executing the statistical methods used in this paper.

## 4. Results and Discussion

In this section, the main results of our research are presented and discussed. In Section 4.1, a comparison of several classifiers by using our proposed database for detecting different pneumatic failures on TIBs is presented. Finally, in Section 4.2, another comparison of all tested classifier by using our proposed database and the databases proposed in [6] for detecting pneumatic failures, as a two-class problem, on TIBs is presented.

To simplify the presentation, a supplementary material website (http://sites.google.com/site/octavioloyola/papers/CP4DPFonTIBs) has been created for this paper, which contains all experimental results, extracted contrast patterns, our proposed database, as well as the fold partitions used in our experiments, and all statistical test results.

### 4.1. Comparing Classifiers for Detecting Different Pneumatic Failures on TIBs

For this comparison, we used the classifiers outlined in Section 3.2 but we excluded those following a one-class approach (B-TPMiner and OKCRA) because our proposed database is a multi-class database; hence, one-class classifiers cannot work there.

Table 4 shows the average AUC results obtained by the tested classifiers in our proposed database for detecting different pneumatic failures on TIBs. In this table, results on the diagonal represent the average AUC result regarding each tested classifier. In addition, this table contains the differences among all classifiers where absolute values greater than 0.05 are considered as an important difference, and, as a consequence, their values are highlighted with an asterisk (*) symbol.

In Table 4, we can see that all classifiers, except AB.M1, obtained an average AUC result above 0.94. In addition, it can be noticed that RF, MLP, TreeBagger, and PBC4cip have an average AUC result close to 1 without important differences among them. Nevertheless, among these classifiers with high average AUC results, PBC4cip provides a model that is close to the language used by human experts in the application domain. Hence, based on this reason, we suggest using PBC4cip for detecting different pneumatic failures on TIBs.

These above-mentioned results are not significantly conclusive because there are several classifiers and only one database, and, as a consequence, no statistical test can be applied. Hence, we performed another experiment where we used the collection of databases introduced in [6] and also converted the database used in this experiment into a two-class problem. By doing this, we used statistical test to corroborate the following: (i) if classifiers reporting good classification results in these experiments can detect failures they were not trained for; and (ii) if the pattern-based approach continues obtaining good classification results.

### 4.2. Comparing Classifiers for Detecting One Type of Pneumatic Failure on TIBs

In the previous section, we show that several classifiers achieved more than 0.99 of AUC when classifying failures they were already trained for. In this section, we test the performance of the classifiers for recognizing new types of failures. Hence, we created six new databases that simulates the occurrence of new types of failures. For every failure type described in Section 3.1, we included the objects of the failure in the testing dataset and included the objects labeled with a different failure in the training dataset. Consequently, every classifier trains with failures different to those it must recognize. In this way, we can corroborate if the tested classifier is able to recognize new types of failures.

**Figure 6:**
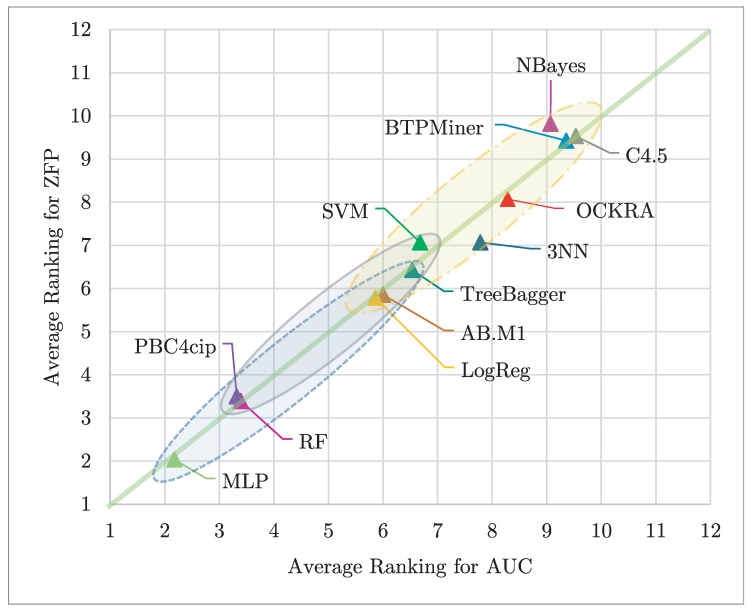
Average ranking for the tested classifiers, according to AUC vs ZFP. Those results closest to the origin (1,1) are the best considering both performance metrics; in addition, those enclosed in an ellipse have no statistical differences among them regarding both evaluated measures.

Figure 6 shows a scatter plot of the average Friedman ranks, according to AUC and ZFP, for all tested classifiers. In this figure, the best classifiers according to ZFP appear at the bottom and the best classifiers according to the AUC appear on the left. Hence, the classifier closest to the origin (1,1) is the best considering both performance metrics. In addition, in this figure, those classifiers enclosed into an ellipse have no statistical differences among them regarding both evaluated measures. In Figure 6, we can see that MLP, PBC4cip, RF, LogReg, AB.M1, and TreeBagger, in this order, obtained better ranking positions, for both AUC and ZFP, than the remaining tested classifiers. In addition, it can be noticed that MLP, PBC4cip, RF, AB.M1, TreeBagger and LogReg are statistically different respect to the remaining tested classifiers. Nevertheless, it is important to highlight that MLP, PBC4cip, and RF are the only classifiers obtaining ranking results below four for both measures.

**Figure 7:**
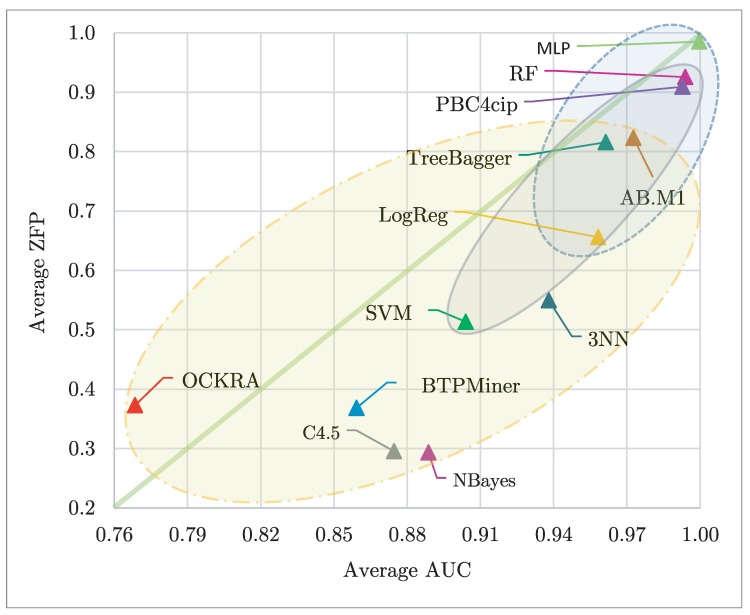
Average AUC vs average ZFP for the tested classifiers. Those results closest to the upper right corner are the best considering both performance metrics; in addition, those enclosed in an ellipse have no statistical differences among them regarding both evaluated measures.

Figure 7 shows a scatter plot of the average classification results, according to AUC and ZFP, for all the tested classifiers. In this figure, the best classifiers according to ZFP appear at the top, and the best classifiers according to AUC appear at the right. Hence, the classifier closest to the upper right corner is the best one considering both performance metrics. Additionally, in this figure, those classifiers enclosed into an ellipse have no statistical differences among them regarding both evaluated measures. In Figure 7, we can see that MLP, RF, PBC4cip, AB.M1, TreeBagger, and LogReg, in this order, obtained the best classification results, for both average AUC and average ZFP, regarding the remaining tested classifiers. In addition, it can be noticed that MLP, PBC4cip, and RF obtained values above 0.90 for both metrics.

From our results, we can conclude that MLP, PBC4cip, and RF obtained the best classification results for detecting pneumatic failures on TIBs. Nevertheless, from these classifiers, PBC4cip has an additional advantage because it provides an explanatory model, which is close to the language used by experts in the application domain.

### 4.3. Analyzing the Extracted Contrast Patterns

In Table 5, we show, for each class, an example of a contrast pattern extracted from our proposed database by using PBC4cip. In this table, for each class (Class), we show a pure (a contrast pattern is pure when it covers objects from only one class [18,59,60]) contrast pattern (Contrast Pattern) and its support (*Supp*). In Table 5, we can see that most of the contrast patterns have items containing the new features proposed by us, which contain the variations of air pressure. In this way, we corroborate that our feature representation helps to obtain useful contrast patterns, with high support, which describe the different types of failures that could arise on a TIBs. In addition, in this table, we can see that there are contrast patterns having only a few items, which are easier to understand by experts. For example, from the first pattern in this table ([Pfv5≤0.02]∧[deltaPs70>0.00]), we can interpret the following: if at 5 s of initialized the immersion time the air pressure into the liquid medium container is lower of equal than 0.02, and at the end of the immersion time the variation of air pressure is greater than 0 then there is a failure of type 1 (fault 1), which means that there is pressure failure on the fitting of the siphon into the liquid medium container.

Table 6 shows, for each class, an example of a contrast pattern extracted from each database described in Section 3.1 but using PBC4cip. In this table, for each database (DB) and each class (Class), we show a pure contrast pattern (Contrast Pattern) and its support (Supp). In this table, we can notice that all contrast patterns have a high support, above 85%, and they have at most three items, which is good because, commonly, those patterns having a few items and high support, provide better classification results and are easier to understand by experts. In this table, we can see that the feature Ps10 is very important for detecting failures on TIBs. Notice that Ps10 is contained in several contrast patterns, and as a consequence, together with the pneumatic expert, we have extracted the following rule to be introduced into the PLC: IF Ps10≤0.13 THEN Failure. This rule will warn about a fault just 10 s after starting any pneumatic action and the main reason is an air leak in the central line of the pneumatic system.

Table 7 shows, for each class, an example of a contrast pattern extracted from our proposed database by using PBC4cip but using our database as a two-class problem as we stated in Section 3.1. In this table, for each two-class database (DB) and each class (Class), we show, for each database and each class, a pure contrast pattern (Contrast Pattern) and its support (Supp). In this table, we can see that most of the contrast patterns have items containing the new features proposed by us in Section 3.1. In this way, we can corroborate that our feature representation helps to obtain good contrast patterns, with high support, which describe the different types of failures appearing on TIBs, when our proposed database is converted in different two-class problem databases.

In Table 7, we can see that, in this case, the contrast patterns have fewer items than those contrast patterns shown in Table 5, which were extracted from our proposed multi-class database. In addition, the contrast patterns showed in Table 7 have higher support than those in Table 5.

It is important to highlight that Table 5, Table 6 and Table 7 are a random subsample of all contrast patterns extracted from the databases used in this study, but we have analyzed all results and the explanations provided in this section are consistent with all the extracted contract patterns, which are provided in our supplementary material.

### 4.4. Analyzing Classification Results Jointly with Experts

We analyzed the classification results obtained by MLP, PBC4cip, and RF jointly with experts in biotechnology and pneumatic systems. From these interactions, we have obtained the following know-how:

Biotechnology experts: The average AUC results obtained by MLP, PBC4cip, and RF were above 0.98, and the average ZFP results were above 0.9. This means that, of the 16 daily immersions (see Section 2), experts will receive at most two (1.6) false failure alerts. As ZFP measures the number of true failures (TP) when no false failures (FP) is detected (see Section 3.3), then a result of ZFP = 0.9 means a FP = 0.1 (or 1.6 FP from 16 daily immersions). On the other hand, biotechnology experts prefer PBC4cip as the classifier to be used for detecting pneumatic failures on TIBs because this classifier provides contrast patterns describing where the failure arose (plant or liquid medium container, or the central air line). Although MLP and RF have slightly better average AUC and ZFP than PBC4cip, biotechnology experts prefer to use PBC4cip because it also provides specific information from when the failure arises (i.e., if it arises during the immersion time and the specific time interval). Finally, experts suggest extending this explanatory model to include other valuable information as the number of fresh mass, amount of liquid medium, and number of shoots.

Pneumatic system experts: The explanatory model proposed by PBC4cip is very helpful because it obtains high classification results and provides a model based on contrast patterns, which can be converted into rules. These rules can be introduced, in an easy way, into a PLC used to manage the pneumatic system on a TIB. Although MLP obtains better classification results than PBC4cip, the pneumatic system experts comment that the model provided by MLP is very hard to be introduced into a PLC, and it does not provide an explanatory model to detect when and why the pneumatic system of a TIB fails. On the other hand, RF provides a model that could be converted into rules, but it contains significantly more rules than those extracted from the PBC4cip model, and there is not a statistical difference between the classification results obtained by these classifiers.

From this analysis with experts in the application domain, we can conclude that PBC4cip is the best classification model to be used for detecting pneumatic failures on TIBs because it obtains high-quality classification results and also provides a model easy to understand by experts and suitable to be included into a PLC.

## 5. Conclusions and Future Work

In this paper, we propose an approach based on contrast patterns for detecting pneumatic failures on TIBs. Our proposal obtained significantly better classification results than other state-of-the-art classifiers, on micropropagation pineapple databases, for detecting pneumatic failures on TIBs. In addition, our proposal obtained the best classification results together with Random Forest and Multilayer Perceptron but our proposal provided a model that is easier to understand by experts and suitable to be included into a programmable logic controller.

On the other hand, we introduced a feature representation for this kind of problems and a new micropropagation pineapple database for detecting four new type of pneumatic failures on TIBs. In addition, we provided a joint analysis with both biotechnology and pneumatic experts regarding the classification results obtained by the classifiers that produced the best results.

As every research, this one can be improved. First, although we used a filtering method for obtaining a set of high-quality patterns covering all instances in the training dataset, this set is a bit strong to be interpreted by a human expert. Second, this research was focused on detecting pneumatic failures on TIBs, but there are other features for obtaining high-quality plants on TIBs, which are important for experts in biotechnology.

Based on these possible improvements, we suggest the following lines for future work. First, new algorithms for discovering patterns could be proposed for extracting a small set of high-quality patterns. Second, a new filtering method for patterns can be proposed for detecting pneumatic failures. Finally, by using methods for processing images, coming from both plant and medium liquid container, experts in biotechnology can obtain valuable information about the micropropagation procedure.

## Figures and Tables

**Table 1:** Description of our micropropagation pineapple database, proposed in this paper, for detecting pneumatic failures on TIBs.

Class	Class_Description	#Objects
fault 1	Pressure failure on the fitting of the siphon for both liquid medium and plant container	72
fault 2	Pressure failure on the fitting of the filter for both liquid medium and plant container	156
fault 3	Pressure failure on the fitting of the siphon into the plant container	83
fault 4	Pressure failure on the fitting of the siphon into the liquid medium container	81
fault 5	Pressure failure on the fitting of the filter into the plant container	129
fault 6	Pressure failure on the fitting of the filter into the liquid medium container	117
normal	The pressure is normal for the pneumatic system on TIBs	313

**Table 2:** Summary of micropropagation pineapple databases for detecting pneumatic failures on TIBs, which were introduced in [6].

Name	#Objects_Min	#Objects_Maj	IR
Pineapple 1	17	236	13.88
Pineapple 2	11	236	21.45
Pineapple 3	25	237	9.48
Pineapple 4	14	237	16.93
Pineapple 5	19	236	12.42
Pineapple 6	16	237	14.81
Pineapple 7	15	237	15.80
Pineapple 8	12	234	19.50

**Table 3:** Supervised classifiers selected in this paper for testing the performance in the detection of a pneumatic failure in a TIB.

Abbrev.	Name	Approach	Ref.
kNN	K-Nearest Neighbor	Based in distance among the objects	[39]
AB.M1	Adaptive Boosting	Boosting	[40]
C4.5	Decision tree	Decision tree	[41]
LogReg	Logistic Regression	Regression	[42]
MLP	Multilayer Perceptron	Neural network	[43]
NBayes	Naïve Bayes	Probabilistic	[44]
RF	Random Forest	Decision trees	[45]
SVM	Support Vector Machine	Support Vector Machine	[46]
TreeBagger	TreeBagger	Bootstrap	[47]
PBC4cip	Pattern-based Classifier for Class Imbalance Problems	Pattern-based classification	[17]
B-TPMiner	Bagging-TPMiner	One-class classification	[48]
OCKRA	One-Class K-means with Randomly-projected features Algorithm	One-class classification and unsupervised classification	[49]

**Table 4:** Average AUC results obtained by the tested classifiers in our proposed database.

Classifier	SVM	RF	MLP	LogReg	C4.5	NBayes	TreeBagger	AB.M1	3NN	PBC4cip
**SVM**	**0.9410**	−0.0554 *	−0.0552 *	0.0004	−0.0433	−0.0168	−0.0496	0.6623 *	−0.0406	−0.0504 *
**RF**		**0.9964**	0.0002	0.0558 *	0.0121	0.0386	0.0058	0.7177 *	0.0148	0.0050
**MLP**			**0.9962**	0.0555 *	0.0119	0.0384	0.0056	0.7175 *	0.0146	0.0048
**LogReg**				**0.9406**	−0.0436	−0.0172	−0.0499	0.6620 *	-0.0410	−0.0507 *
**C4.5**					**0.9843**	0.0264	−0.0063	0.7056 *	0.0026	−0.0071
**NBayes**						**0.9578**	−0.0327	0.6791	−0.0238	−0.0336
**TreeBagger**							**0.9906**	0.7119 *	0.0089	−0.0008
**AB.M1**								**0.2787**	−0.7029 *	−0.7127 *
**3NN**									**0.9816**	−0.0098
**PBC4cip**										**0.9914**

**Table 5:** Examples of contrast patterns extracted from our proposed database by using PBC4cip.

Class	Contrast Pattern	Supp
fault 1	[Pfv5≤0.02]∧[deltaPs70>0.00]	85%
fault 2	Pfv8>0.00]∧[Pfv54≤0.12]∧[Ps26≤0.10]∧[Ps7≤0.08]∧[Pfll11≤0.06]	49%
fault 3	[Ps48>0.10]∧[deltaPs33≤0.00]∧[Ps48≤0.18]∧[deltaPfv70≤−0.02]∧[Ps7≤0.16]∧[Ps65>0.10]	39%
fault 4	[Ps35≤0.20]∧[Pfv13>0.00]∧[Ps46≤0.13]∧[Ps44≤0.07]∧[deltaPfll2≤0.01]∧[deltaPs32≤0.00]∧[Pfv7≤0.02]	40%
fault 5	[Pfv34≤0.02]∧[Ps58≤0.10]∧[Ps48>0.10]	44%
fault 6	[Pfv8≤0.00]	67%
normal	[Ps51>0.10]∧[Pfv24>0.02]∧[Pfll47>0.02]∧[Ps5>0.10]	80%

**Table 6:** Examples of contrast patterns extracted from the databases proposed in [6] by using PBC4cip.

DB	Contrast Pattern	Class	Supp
BITPinepple 1	[Ps(48)>0.10]∧[Pfll(1)≤0.11]	normal	86%
	[Ps(45)≤0.11]∧[Pfll(13)>0.11]∧[Ps(41)>0.08]	failure	86%
BITPinepple 2	[Pfll(1)>0.07]∧[Pfv(59)≤0.11]	normal	80%
	[Ps(55)≤0.12]∧[Pfv(59)>0.11]∧[Pfll(62)≤0.02]	failure	100%
BITPinepple 3	[Ps(1)≤0.09]	normal	88%
	[Pfll(4)≤0.12]∧[Ps(41)>0.09]	failure	100%
BITPinepple 4	[Ps(20)≤0.11]	normal	84%
	[Ps(20)>0.11]∧[Pfll(8)≤0.14]	failure	100%
BITPinepple 5	[Pfll(12)>0.12]	normal	85%
	[Pfv(46)>0.12]∧[Ps(10)≤0.12]	failure	100%
BITPinepple 6	[Pfll(17)>0.11]	normal	86%
	[Ps(14)≤0.11]∧[Ps(42)>0.10]	failure	100%
BITPinepple 7	[Ps(43)≤0.13	normal	80%
	[Ps(54)>0.13]∧[Ps(10)≤0.13]	failure	92%
BITPinepple 8	[Pfv(42)≤0.13]	normal	86%
	[Ps(10)≤0.12]∧[Pfv(54)>0.13]	failure	100%

**Table 7:** Examples of contrast patterns extracted from our proposed database, as two-class problems, by using PBC4cip.

DB	Contrast Pattern	Class	Supp
Fault 1	[deltaPs2>0.00]∧[Pfv2>0.02]	normal	69%
	[Pfv26≤0.02]∧[Ps31>0.15]∧[Pfv33≤0.02]	failure	75%
Fault 2	[Pfv31>0.02]∧[Pfv37>0.02]	normal	78%
	[Pfv37≤0.02]∧[Ps25>0.07]∧[deltaPfll3≤0.00]	failure	79%
Fault 3	[Pfv22>0.02]∧[deltaPfll2>0.00]	normal	76%
	[Pfll44≤0.02]∧[Pfv34≤0.02]∧[Pfv68>0.06]	failure	64%
Fault 4	[Pfv15>0.02]∧[Ps11>0.08]	normal	77%
	[Pfv23≤0.02]∧[Ps53>0.08]∧[deltaPs60>0.00]	failure	75%
Fault 5	[Pfv9>0.02]∧[deltaPs42>0.00]∧[Pfv19>0.01]	normal	80%
	[Pfll35≤0.02]∧[Pfv30≤0.01]	failure	55%
Fault 6	[deltaPfll2>0.01]∧[Pfv4>0.02]	normal	78%
	[Pfll68≤0.02]∧[Ps67>0.06]∧[Pfv15≤0.02]	failure	60%

## Abbreviations

The following abbreviations are used in this manuscript:

AB	Adaptive Boosting
AUC	Area Under the receiver operating characteristic Curve
B-TPMiner	Bagging-TPMiner
CP	Contrast Pattern
DB	Database
kNN	K-Nearest Neighbor
MLP	Multilayer Perceptron
NBayes	Naïve Bayes
LogReg	Logistic Regression
PBC4cip	Contrast Pattern-Based Classifier for Class Imbalance Problems
RF	Random Forest
Supp	Support
SVM	Support Vector Machine
TIB	Temporary Immersion Bioreactor
ZFP	Zero-False Positives
